# Multiple DMARD discontinuations in rheumatoid arthritis: how often and in what patients? Results from a national clinical RA register

**DOI:** 10.1136/rmdopen-2025-005617

**Published:** 2025-10-15

**Authors:** Emma Wettersand, Daniela di Giuseppe, Johan Askling, Katerina Chatzidionysiou

**Affiliations:** 1 Karolinska Institute Department of Medicine Solna, Stockholm, Sweden; 2 Clinical Epidemiology Unit, Department of Medicine Solna, Karolinska Institutet, Stockholm, Sweden

**Keywords:** Rheumatoid Arthritis, DMARD, Biological Therapy

## Abstract

**Objectives:**

Multiple discontinuations of biologic (b-) or targeted synthetic (ts-) disease-modifying antirheumatic drugs (DMARDs) may indicate difficult-to-treat disease. We aimed to assess the occurrence of b/tsDMARD discontinuations in patients with rheumatoid arthritis (RA), specifically how this varies by definition, across patient subsets and over time.

**Methods:**

Observational cohort study using data from the Swedish Rheumatology Quality Register on patients diagnosed with RA between 2010 and 2019. We identified three populations: (1) newly diagnosed (N=17 780), (2) initiating a first-ever DMARD (N=18 038) and (3) initiating a first-ever b/tsDMARD (N=8075). In each, we assessed the proportions and characteristics of patients fulfilling either of seven alternative DMARD discontinuation criteria (each encompassing a unique combination of number and type(s) of b/tsDMARD).

**Results:**

At 4.5 years of follow-up, 10% in populations (1) and (2), and 25% in (3), had discontinued ≥2 b/tsDMARDs with different modes of action. The proportions meeting each of the other six definitions ranged from 0.3% to 10% in (1) and (2), and 1% to 25% in (3). Regardless of definition or time, the characteristics of discontinuers across populations (1) through (3) remained largely similar.

**Conclusions:**

Applying treatment discontinuation-based definitions to an unselected RA population identifies widely varying proportions of patients with largely similar characteristics. Quantitatively, treatment-based definitions, follow-up time and study population must be clearly specified; qualitatively, the specific definition may be less critical.

**WHAT IS ALREADY KNOWN ON THIS TOPIC**

**WHAT THIS STUDY ADDS**

- The proportion of DMARD discontinuers ranged from 0.3% to 25%, depending on the definition and the RA population. For instance, within four and a half years from RA diagnosis, 10% of all RA patients failed two or more biological or targeted synthetic DMARDs (b/tsDMARDs) with different mechanisms of action (MoA), while among those starting a first b/tsDMARD, 25% failed two or more b/tsDMARDs MoA within four and a half years.
- Despite the wide range in the proportions of multiple DMARD discontinuations, and regardless of their exact definition, the patient- and disease-characteristics of discontinuers were similar: younger age, female gender, higher Patient Global Assessment, Disease Activity Score with 28 joint count, tender joint count, pain and fatigue.

**HOW THIS STUDY MIGHT AFFECT RESEARCH, PRACTICE OR POLICY**

- Applying definitions based on b/tsDMARD discontinuations to an unselected RA population will identify vastly different proportions of patients but with reasonably similar characteristics.
- From a quantitative point of view, and for interpretable results, not only the treatment-based definition but also time of follow-up and the population must be well-defined. From a qualitative point of view, the exact choice of definition may be less important.

## Introduction

 Thanks to breakthroughs in earlier diagnosis, pharmacological treatment options, treat-to-target (T2T) approaches and otherwise improved treatment strategies, the treatment outcomes of rheumatoid arthritis (RA) have improved markedly over the past few decades.^1^,^4^ However, a significant proportion of patients with RA will accrue multiple discontinuations of disease-modifying antirheumatic drugs (DMARDs). Such patients may constitute a ‘difficult-to-treat’ (D2T) subgroup of RA patients, in need of early identification and tailored clinical intervention. In fact, D2T RA is currently one of the most important unmet clinical needs in the field of RA.

D2T RA is a criteria-based definition of a (presumably heterogeneous) clinical entity. In 2020, EULAR presented a definition of D2T RA according to which D2T RA requires the failure of at least two biological or targeted synthetic DMARDs (b/tsDMARDs) with different mechanisms of action (MoA).^5^ As an umbrella term, D2T RA includes many different subgroups, such as patients with intrinsically refractory RA who exhibit primary lack of effectiveness to multiple DMARDs, patients who experience loss of effectiveness for pharmacological reasons (eg, antidrug antibodies, suboptimal dosing) and patients with remaining pain despite adequate control of concurrent inflammation (eg, concomitant osteoarthritis or pain sensitisation).^6^

In a study from the UK on patients with RA starting first-line tumour necrosis factor inhibitor (TNFi) in the British Society for Rheumatology Biologics Register for RA from 2001 to 2014, 6.4% of the patients started their third class of bDMARD within 8 years and, among those, one out of five continued to a fourth class of bDMARD.^7^ Since the introduction of EULAR’s D2T RA definition, its prevalence has been studied in different RA populations, with several studies reporting that 10%–20% fail at least two b/tsDMARDs with different MoA. Importantly, few of these studies have presented the median time until fulfilment of the D2T definition, neither from RA diagnosis nor from the start of the first b/tsDMARD.^8^,^12^ How b/tsDMARD failures accrue over time and how they vary with patient characteristics and treatment history is thus less well known but essential for a clinically and aetiologically relevant definition of D2T, and for the identification of such patients in clinical practice.

The aim of this study was therefore to investigate how the observed proportions of b/tsDMARD discontinuations, suggestive of D2T RA, are impacted by: (1) different definitions of accrued treatment discontinuations, (2) the choice of the target population, (3) the time-frames under study, and to explore the characteristics of patients meeting these definitions.

## Methods

### Study design

We performed an observational cohort study in patients newly diagnosed with RA between January 2010 and December 2019, based on data from the Swedish Rheumatology Quality register (SRQ), which has an estimated coverage of around 87% of all Swedish patients with RA.^13 14^ The study period was selected to ensure that all non-TNFi bDMARDs were available for prescription in clinical practice. To be eligible, patients had to have at least 24 months of available follow-up time in the register.

### Data collection          

Prospectively collected data on demographics (age, gender), clinical characteristics (symptom duration, smoking status, disease activity) and Patient-Reported Outcomes (PROs) [fatigue, pain, Health Assessment Questionnaire (HAQ), patient global] at baseline were retrieved from SRQ. Seropositivity was defined through International Classification of Diseases 10th revision codes registered in SRQ.

Information on use of csDMARD (yes/no), such as methotrexate, sulfasalazine and leflunomide, use of glucocorticoids (yes/no), time from RA diagnosis to start of first csDMARD and time to start of first b/tsDMARD was collected. Comorbidity data (covering the period 5 years prior to baseline) were collected through linkage to the National Patient Registry and to the Cancer Registry, see online supplemental table 6.

### Patients

We defined three patient populations, with each subsequent population being a subset of the previous one: population (1) included all patients newly diagnosed with RA. Population (2) consisted of those patients from population (1) who initiated their first-ever DMARD, most commonly a conventional synthetic DMARD (csDMARD), such as methotrexate; population (3) comprised patients from population (2) who went on to initiate their first-ever b/tsDMARD during the study period. Importantly, the baseline (start of follow-up) was defined differently for each population to reflect key points in the disease course: for population (1), baseline was set at the time of RA diagnosis. For population (2), baseline was the initiation of the first-ever DMARD. For population (3), baseline corresponded to the initiation of the first-ever b/tsDMARD.

In each population, patients without a baseline visit (defined as a visit −30; +30 days around the baseline) were excluded. A second visit listing RA was required for study inclusion, to guarantee a correct RA diagnosis and treatment start (table 1).

**Table 1:** Flow chart of number of patients in the three patient populations (1/2/3) investigated for the occurrence of repeated discontinuation of b/tsDMARDs, baseline* demographics and patient characteristics

Population (N)	(1) (26 469)	(2) (25 010)	(3) (11 132)
Excluded patients without a second visit counted from baseline*	8490	6760	2823
We excluded all patients without the following ICD codes: M05, M06.0L, M06.8L, M06.0, M06.0M, M06.0N, M06.8M, M06.8N and M06.9	186	187	189
Patients younger than 18 years were excluded from the analysis	13	25	45
Excluded patients (N)	8689	6972	3057
Included patients (N)	17 780	18 038	8075

* Baseline definitions (1) date of RA diagnosis (2) start date first ever DMARD (3) start date first ever b/tsDMARD.

b/tsDMARDs, biological or targeted synthetic DMARDs; DMARDs, disease-modifying antirheumatic drugs; ICD, International Classification of Diseases.

### Definitions of multiple b/tsDMARD treatment discontinuation

We devised seven treatment-based definitions of discontinuation of different numbers and types of b/tsDMARDs (A–E). In each population (1)–(3), we investigated the proportion and timing of fulfilment of each definition (table 2):

**Table 2:** The treatment-based definitions of repeated discontinuation of b/tsDMARDs

Definition	Number of b/tsDMARDs discontinued	Type of b/tsDMARDs
A	≥2	At least two bDMARDs with different MoA
A1	≥2	At least one tumor necrosis factor inhibitor (TNFi) (infliximab and/or etanercept and/or adalimumab and/or certolizumab and/or golimumab) AND at least one non-TNFi (rituximab and/or abatacept and/or interleukin 6 (IL-6) inhibitors (tocilizumab or sarilumab)) bDMARD
A2	≥2	At least two non-TNFis with different MoA
B	≥3	At least three bDMARDs with different MoA
B1	≥3	At least one TNFi (infliximab and/or etanercept and/or adalimumab and/or certolizumab pegol and/or golimumab) AND two non-TNFis with different MoA
B2	≥3	At least three non-TNFis with different MoA
C	≥4	At least one TNFi and all available non-TNFis
D	≥5	At least one TNFi and all available non-TNFis PLUS at least one tsDMARD
E	≥2	≥2 b/tsDMARDs with different MoA

bDMARD, biological disease modifying antirheumatic drug; b/tsDMARDs, biological or targeted synthetic DMARDs; DMARDs, disease-modifying antirheumatic drugs; MoA, mechanisms of action; TNFi, tumour necrosis factor inhibitor; tsDMARD, targeted synthetic modifying antirheumatic drug.

A1+A2: failure of at least two bDMARDs.

B1+B2: failure of at least three bDMARDs.

C: failure of at least one TNFi and all available non-TNFis.

D: failure of at least one TNFi and all available classes of non-TNFis plus at least one tsDMARD.

E: failure of ≥2 b/tsDMARDs with different MoA.

We considered the following MoAs in b/tsDMARDs for RA: TNFi, B-cell depletion, T-cell co-stimulation modulation, interleukin 6 inhibition and JAK inhibition.

For the seven definitions A–E, we did not set any time-limits for: the duration from discontinuation of one b/tsDMARD until the start of the next, the duration of each treatment, the time from RA diagnosis until the first b/tsDMARD start, nor did we pay attention to the stated reason for the discontinuation of each b/tsDMARD.

In sensitivity analyses, we used the same definitions A–E but with restraints on time in order to define a group of treatment-refractory RA: the time from RA diagnosis until the start of the first b/tsDMARD was capped at 2 years, the time on each treatment was set to a maximum of 12 months, and the time between discontinuation of one b/tsDMARD until the start of the next b/tsDMARD had to be less than 6 months (online supplemental figure 2). We applied no restriction based on the reason for treatment discontinuation.

For all definitions and outcomes, switching from an originator bDMARD drug to its biosimilar product, from one biosimilar product to another or back to the originator bDMARD was not considered as a change of treatment. Similarly, switching back to a previous DMARD after failure of one or more other DMARDs was not considered switching.

### Statistical analyses

We assessed the proportions of patients, in each of population (1), (2) and (3), who fulfilled each of the definitions A–E and calculated the cumulative incidences of these proportions at 5 years, counting from the baseline in each population.

Continuous variables are presented as mean±SD for normally distributed variables and medians (with IQRs) for non-normally distributed variables. Categorical variables are presented as percentages. We assessed baseline characteristics in each of the populations (1), (2) and (3). Since definition E is the one that corresponds to EULAR D2T criterion 1, we chose to present this definition in the text (see table 2 and supplementary table 1 for definitions A–D). Smoking status and evaluator global assessment of disease activity (EGA) were missing in over 50% of cases and were not analysed. Missing data in all three nested patient populations are shown in online supplemental table 5.

We investigated any calendar time effect on the outcomes (fulfilment of each definition) and patient characteristics at baseline in all three patient populations through stratification of the study period into two intervals (2010–2014 and 2015–2019). We additionally performed analyses with follow-up time limited to 4 years to make the available follow-up time in the two calendar period strata comparable.

All analyses were performed using SAS V.9.4 (SAS Institute, Cary, North Carolina, USA) and IBM SPSS Statistics V.25 software.

## Results

**Figure 1:**
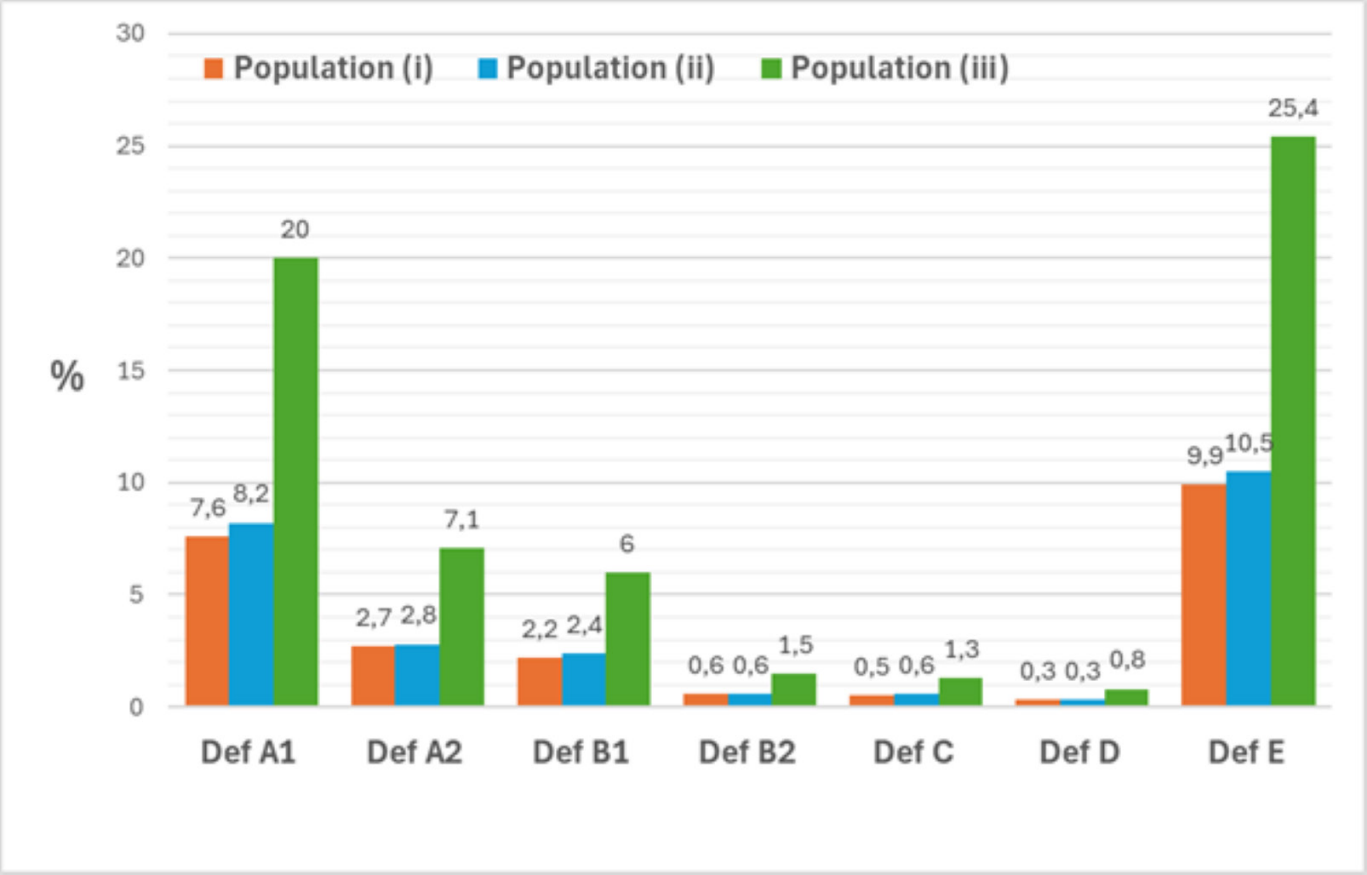
Proportions of patients fulfilling the definitions (**A–E**) in the three patient populations (population (1), (2) and (3)).

*Proportions fulfilling DMARD discontinuation definitions A to E in the three populations*. We identified 17 780 patients with newly diagnosed RA in population (1). Baseline characteristics are summarised in table 3. During a median of 93 (IQR: 61–122) months of follow-up, the percentage of patients fulfilling definitions A–E ranged from 0.3% to 10% (figure 1 and online supplemental table 1). The median (IQR) time from RA diagnosis until the above outcomes were fulfilled ranged from 25 (12–47) to 80 (64–96) months. In the sensitivity analysis with time restrictions taken into consideration, much fewer patients fulfilled the same definitions (0.03%–2.2%, online supplemental table 2).

**Table 3:** Baseline characteristics of the three patient populations (1), (2) and (3)

Baseline characteristics*	Population (1)		Population (2)		Population (3)	
	All patients	Patients fulfilling definition E	All patients	Patients fulfilling definition E	All patients	Patients fulfilling definition E
N (with at least one following visit after baseline visit)	17 780	1752 (9.9%)	18 038	1895 (10.5%)	8075	2047 (25.4%)
Age (years), mean (SD)	60.5 (15.1)	53.5 (14.1)	59.8 (15.1)	53.2 (14.2)	54.2 (14.8)	53.1 (14.2)
Sex (male), %	30.7	21.8	30.8	22.8	26.8	23.0
Symptom duration before RA diagnosis, months, median (IQR)	9 (4–46)	11 (4–57)	8 (4–30)	9 (4–40)	11 (4–50)	11 (4–47)
CRP, mg/L, median (IQR)	9 (4–22)	10 (4–26)	9 (4–22)	10 (4–25)	7 (3–18)	8 (4–21)
ESR, mm/h, median (IQR)	23 (12–40)	24 (12–41)	23 (12–40)	22 (12–40)	19 (10–34)	20 (10–36)
PGA, VAS 0–100, mean (SD)	49.0 (26.7)	57.5 (25.8)	49.4 (26.3)	57.1 (25.5)	53.2 (24.7)	57.4 (23.5)
Pain, VAS 0–100, mean (SD)	50.5 (26.7)	58.6 (26.0)	50.8 (26.4)	58.3 (25.7)	53.8 (25.2)	58.2 (24.1)
Fatigue, VAS 0–100, mean (SD)	48.9 (28.7)	58.9 (27.9)	49.1 (28.4)	58.9 (27.5)	53.3 (27.3)	58.5 (26.0)
HAQ, mean (SD)	0.97 (0.66)	1.13 (0.66)	0.97 (0.65)	1.12 (0.66)	0.98 (0.63)	1.10 (0.63)
SJC, median (IQR)	5 (2–9)	6 (3–10)	5 (2–9)	6 (3–10)	4 (2–8)	5 (2–9)
TJC, median (IQR)	5 (2–9)	6 (3–11)	5 (2–9)	6 (3–11)	5 (2–9)	6 (3–10)
Concomitant csDMARD, %	81.0	79.0	94.4	85.2	74.2	72.3
Concomitant GC, %	46.5	48.6	53.5	56.0	45.4	54.6
RF and/or anti-CCP pos (%)	71.0	79.6	71.1	79.1	77.5	78.1
DAS-28-ESR, mean (SD)	4.65 (1.50)	5.1 (1.4)	4.7 (1.5)	5.0 (1.4)	4.6 (1.3)	4.9 (1.2)
Comorbidities at baseline						
Heart failure, %	1.4	0.7	1.3	0.7	1.0	1.1
Ischaemic heart disease, %	4.0	2.3	3.8	2.7	3.0	3.1
Malignancy, %	3.4	1.7	3.3	1.6	1.8	2.0
Renal failure, %	0.5	0.3	0.5	0.4	0.6	0.3
COPD, %	1.6	1.6	1.6	1.8	1.6	1.7
All respiratory, %	12.2	14.4	12.0	14.2	14.7	15.4
Hospital infections, %	5.6	5.1	5.5	5.4	6.7	6.3

* Baseline definitions (1) date of RA diagnosis (2) start date first-ever DMARD (3) start date first ever b/tsDMARD.

bDMARD, biological disease modifying antirheumatic drug; CDAI, clinical disease activity index; COPD, chronic obstructive pulmonary disease; CRP, C reactive protein; csDMARD, conventional synthetic disease modifying antirheumatic drug; DAS-28, Disease Activity Score-28; ESR, erythrocyte sedimentation rate; GC, glucocorticoids; HAQ, Health Assessment Questionnaire; PGA, Patient Global Assessment; RA, rheumatoid arthritis; Seropositive, anti-CCP (cyclic citrullinated peptide) and/or RF (rheumatoid factor) positive; SJC, swollen joint count; TJC, tender joint count; tsDMARD, targeted synthetic modifying antirheumatic drug; VAS, visual analogue scale.

Population (2), defined as patients who started a first-ever DMARD, comprised 18 038 patients (table 3). During a median follow-up of 89 (57–121) months, 0.3%–10% of patients fulfilled definitions A–E (figure 1 and online supplemental table 1). Few patients (0.04%–2.3%) fulfilled the same definitions with time restrictions added (online supplemental table 2). The median (IQR) time from RA diagnosis until the above outcomes were fulfilled ranged from 26 (12–49) to 61 (43–84) months.

Finally, 8075 patients started a first-ever b/tsDMARD (population (3), table 3). During a median follow-up of 68 (36–103) months, 1%–25% of these patients fulfilled definitions A–E (figure 1 and online supplemental table 1). The median (IQR) time from start of the first ever b/tsDMARD until the definition of each outcome was fulfilled ranged from 24 (12–49) to 80 (63–96) months. Fewer patients (0.09%–5.8%) fulfilled the definitions when time restrictions were added (online supplemental table 2).

Notably, for definition E, the median time from baseline to meeting the criteria was 4½ years across all three populations. An overview of the study is presented in online supplemental figure 1.

The 5-year cumulative incidence (%) of outcomes A to E ranged from 0.06% to 5.7% in population (1), 0.2% to 6.0% in population (2) and 0.2% to 14.6% in population (3).

### Patient and disease characteristics

Baseline characteristics of patients fulfilling definition E in the three patient populations are presented in table 3, data on those fulfilling definitions A–E are presented in online supplemental table 3. compared with the whole populations (1) to (3), those patients fulfilling definition E were younger, more often female, more often seropositive, had higher C reactive protein, Patient Global Assessment (PGA), tender joint count (TJC), Disease Activity Score-28 (DAS-28), and were less likely to be treated with concomitant csDMARDs but more likely to be treated with concomitant glucocorticoids at baseline.

### Calendar period and restricted follow-up time

During the 2010–2014 period, the median follow-up was 117 (101–133) months for population (1), 116 (98–133) months for population (2) and 95 (68–120) for population (3). For the period 2015–2019, the median duration of follow-up was 56 (40–70) months for population (1), 53 (38–68) months for population (2) and 38 (22–56) months for population (3). In online supplemental table 7, the proportions of patients in each calendar period fulfilling the definitions A to E, the median time for fulfilment (months) and the cumulative incidence at 5 years from RA diagnosis are presented. The proportions of patients fulfilling the definitions were higher in the first calendar period. However, once follow-up was restricted to 4 years (to enable a fair comparison across calendar periods), we observed more similar proportions with only a tendency towards lower proportions of patients fulfilling the definitions of discontinuation of b/tsDMARDs in the latter calendar period (2015–2019) compared with the earlier one (2010–2014) in all three patient populations (online supplemental table 4).

## Discussion

In this large, real-world observational study based on a large national register of patients with RA, we assessed the proportions fulfilling various definitions of treatment failures defined via DMARD use, in different patient segments and over time. We made several important observations: (1) the proportions fulfilling our outcomes varied widely, from close to 0% up to 25%, with the exact outcome definition, the underlying RA population and time, indicating the importance from a quantitative point of view of defining all three aspects, (2) almost irrespective of definition and population, the patient- and disease-characteristics of those who fulfilled each definition was similar, at least relative to the characteristics of each of the three underlying study cohorts, suggesting that from a qualitative point of view, patients who fulfil definitions of failed treatments share many characteristics in common irrespective of the exact definition of treatment-based failure or line of treatment.

Within a median time of 4.5 years from the RA diagnosis, around 10% of all RA patients (population (1)) fulfilled definition E (here: failure of ≥2 b/tsDMARDs with different MoA, ie, EULAR D2T criterion 1). In our study, among definitions A to E, the proportion of patients fulfilling definition (E) was the highest of the definitions A to E, in all three populations (1) to (3). Similarly, one-fourth of all patients who started a first-ever b/tsDMARD (population (3)) failed at least two subsequent b/tsDMARDs with different MoA, within a median time of 3 years, and fulfilled outcome E. These high proportions have important clinical implications, underscoring the need to optimise ongoing b/tsDMARD therapy before considering a switch, including evaluation of non-primary failure causes of suboptimal response. This may involve a follow-up visit with comprehensive assessment and, when appropriate, a multidisciplinary review involving RA care specialists.

Our results agree with previous studies on D2T RA, both regarding the results from studies on unselected RA populations^8 12 15^ and populations of RA patients starting a b/tsDMARD.^10 16^ With respect to previous studies in incident RA, one study on early stages of RA (up to 5 years after diagnosis) presented a prevalence of D2T of <6%.^17^ We found that the 5-year cumulative incidence of fulfilling the first criterion of the D2T definition in newly diagnosed RA was 2.3 %, but also demonstrated how the exact definition of the patient population and the outcome will make the proportion of patients with at least seemingly difficult-to-treat RA disease vary widely.

By adding clinically reasonable maximum time intervals to the definitions A–E (time between RA diagnosis and start of the first b/tsDMARD, time on treatment, time between one DMARD discontinuation and start of another) according to a T2T paradigm, the proportion of patients who reached our outcome definitions decreased markedly. One possible explanation is that many patients ‘remain’ on a b/tsDMARD for a long period of time, suggesting at least a reasonable effectiveness, and that discontinuation after a longer period is therefore primarily because of late-onset toxicity or secondary loss of effectiveness, or other reasons.

Regarding the potential effect of calendar period, our period-specific analyses with follow-up restricted to 4 years indicated that a slightly lower proportion of patients fulfilled the definitions (A–E) in the later time period (2015–2019). This tendency towards a lower proportion of patients fulfilling multiple b/tsDMARD treatment failure in the recent time period was observed also in some of the previous studies on D2T RA.^7 16^ We hypothesise that improved management strategies with earlier diagnosis, ultrasound-based screening and tight control during the later time period have had an impact on the (somewhat reduced) occurrence of multiple treatment discontinuation despite increasing treatment alternatives.

One of our hypotheses in this study was that there are common clinical and disease characteristics across the different outcome definitions. We could confirm this hypothesis, which suggests that whereas the exact choice of definition will impact the observed prevalence of D2T RA, the exact choice of the definition itself may be less important in terms of the prediction of patients *at elevated risk for* repeated discontinuation of treatments, as a sub-criterion of D2T RA. In our study, certain baseline characteristics were more often observed in patients fulfilling (vs not) the outcome definitions A–E, irrespective of the type of definition that we used. These baseline characteristics were younger age, female sex, higher PGA, TJC and DAS-28. Patients fulfilling definition D were more markedly younger and exhibited higher levels of PGA, pain and fatigue compared with those meeting the other definitions. These characteristics align with definition D, representing the most stringent and symptom-driven criterion among the definitions evaluated.

This observation agrees with previous studies that have shown that female gender, younger age, high disease activity, HAQ, fatigue and pain at baseline and seropositivity are predictors for DMARD treatment failure^7 8^ and fulfilment of D2T RA criteria,^718^,^20^ both at time of diagnosis,^15^ when patients started therapy^10 16^ and at the timepoint of the most recent visit before fulfilment of D2T criteria.^12^ In an early RA cohort with D2T within 5 years, lower physical functioning and worse levels of pain at early stages of the disease emerged as predictors of D2T.^17^ Age could be a contraindication for b/tsDMARD when certain comorbidities are present, so this observation was not surprising but forms an important facet of D2T-RA. Seropositive RA was more common among patients that fulfilled the outcome definitions in our study, seropositivity and in particular high levels of ACPA antibodies a well-established risk factor for worse prognosis and structural damage, hence rheumatologists might be more prone to initiate biological treatment in patients with seropositive RA. Increased attention and closer monitoring of patients with higher disease burden at baseline is thus warranted. Higher pain levels might indicate higher disease activity but may also point to a subgroup of patients with other reasons for remaining pain despite adequate inflammatory control (non-inflammatory refractory RA).

Our study is one of the hitherto largest studies assessing the occurrence of different definitions of b/tsDMARDs discontinuations in RA patient populations. Besides the large study population, strengths include the high external validity due to the nature of data deriving from a real-life, nation-wide register, during a period when all three non-TNFis bDMARDs (abatacept, tocilizumab and rituximab) were available for prescription. We assessed alternative outcome definitions depending on the number and type of different b/tsDMARDs, in three different nested patient populations, which has not been reported before. We included time (on treatment, between one DMARD discontinuation and the start of another), and observed its major impact on numbers. This demonstrates that time is probably an important factor in studies of true intrinsic refractoriness using registry data.

Our study has some limitations, such as non-negligible missingness for some variables (eg, smoking and EGA), which was therefore excluded from further analyses. Data comes from a ‘real-world’ clinical practice, and a physician’s examination and opinion might underestimate disease activity, and excessive restrictiveness regarding treatment in case of comorbidities might affect the results. Under-reporting might also be an issue, leading to a risk of underestimating the occurrence of the outcomes. On the other hand, since we did not include reasons for discontinuation, for example, pregnancy or remission, there is a risk of overestimation of the occurrence of the outcomes.

To summarise, a substantial proportion of patients with RA discontinue b/tsDMARDs. Applying treatment-based definitions of b/tsDMARD treatment failure to an unselected RA population will identify vastly different proportions of patients (0.3%–25%), but with reasonably similar characteristics, suggesting that the exact choice of definition is probably less important from a qualitative but not quantitative point of view, and when it comes to defining individuals at elevated risk of D2T. Our results further underscore the need to define the RA patient population to which treatment-based definitions of D2T are to be applied, with the occurrence of multiple DMARD discontinuation increasing significantly in patients already introduced to a first b/tsDMARD. Finally, our results suggest that increased attention to RA patients with the following characteristics: younger age, female sex, higher PGA, TJC and DAS-28, at the time of diagnosis and when they initiate the first DMARD or b/tsDMARD, may help identify D2T RA.

## Supplementary material

10.1136/rmdopen-2025-005617 online supplemental file 1

10.1136/rmdopen-2025-005617 online supplemental file 2

10.1136/rmdopen-2025-005617 online supplemental file 3

10.1136/rmdopen-2025-005617 online supplemental file 4

10.1136/rmdopen-2025-005617 online supplemental file 5

## Data Availability

Data are available upon reasonable request. All data relevant to the study are included in the article or uploaded as supplementary information.
