# Supplementary material for: Multiple DMARD discontinuations in rheumatoid arthritis: how often and in what patients? Results from a national clinical RA register
Source: RMD Open. 2025 Oct 15;11(4):e005617. doi: 10.1136/rmdopen-2025-005617 (PMC12530398; doi:10.1136/rmdopen-2025-005617)
Supplement: online supplemental file 5 [file rmdopen-11-4-s005.docx]

**Supplementary**

***Supplementary table 1****: Proportions of patients fulfilling the definitions (A-E), median time from baseline until the fulfilment of the respectively sub-definition and the cumulative incidence 5 years from baseline^*^, in the three nested patient populations, respectively.*

|  | Population (i) | Population (ii) | Population (iii) |
| --- | --- | --- | --- |
| **Definition A1 % (N)**  **Failure of at least two bDMARDs with different MoA. At least one TNFi and at least one non-TNFi.** | 7.6 % (1357) | 8.2 % (1475) | 20.0 % (1613) |
| Time from baseline until fulfilling the subdefinition, months, median (IQR) | 37 (19–62) | 37 (18–62) | 37 (18–63) |
| Cumulative incidence 5 years (%) | 5.7 % | 6.0 % | 14.6 % |
| **Definition A2 % (N)**  **Failure of at least two bDMARDs with different MoA. At least two non-TNFis.** | 2.7 % (480) | 2.8 % (513) | 7.1 % (573) |
| Time from baseline until fulfilling the subdefinition, months, median (IQR) | 25 (12–47) | 26 (12–49) | 24 (12–49) |
| Cumulative incidence 5 years (%) | 2.3 % | 2.4 % | 5.9 % |
| **Definition B1 % (N)**  **Failure of at least three bDMARDs with different MoA. At least one TNFi AND two non-TNFis.** | 2.2 % (393) | 2.4 % (424) | 6.0 % (481) |
| Time from baseline until fulfilling the subdefinition, months, median (IQR) | 50 (32–74) | 52 (32–78) | 52 (31–76) |
| Cumulative incidence 5 years (%) | 1.3 % | 1.4 % | 3.4 % |
| **Definition B2 % (N)**  **Failure of three non-TNFis** | 0.6 % (103) | 0.6 % (110) | 1.5 % (119) |
| Time from baseline until fulfilling the subdefinition, months, median (IQR) | 62 (43–85) | 60 (43–82) | 60 (43–84) |
| Cumulative incidence 5 years (%) | 0.3 % | 0.3 % | 0.8 % |
| **Definition C (N)**  **Failure of at least one TNFi and all available non-TNFis** | 0.5 % (91) | 0.6 % (100) | 1.3 % (109) |
| Time from baseline until fulfilling the subdefinition, months, median (IQR) | 60 (43–85) | 61 (43–84) | 60 (43–84) |
| Cumulative incidence 5 years (%) | 0.3 % | 0.3 % | 0.7 % |
| **Definition D % (N)**  **Failure of at least one TNFi and all available non-TNFis PLUS at least one tsDMARD** | 0.3 % (53) | 0.3 % (59) | 0.8 % (64) |
| Time from baseline until fulfilling the subdefinition, months, median (IQR) | 80 (64–96) | 61 (43–84) | 80 (63–96 |
| Cumulative incidence 5 years (%) | 0.06 % | 0.2 % | 0.2 % |
| **Definition E % (N)**  **Failure of ≥2 b/tsDMARDs with different MoA** | 9.9 % (1752) | 10.5 % (1895) | 25.4 % (2047) |
| Time from baseline until fulfilling the subdefinition, months, median (IQR) | 54 (31–79) | 53 (31–80) | 53 (30–79) |
| Cumulative incidence 5 years (%) | 2.3 % | 2.4 % | 5.8 % |

** Baseline definitions i) date of RA diagnosis ii) start date first ever DMARD iii) start date first ever b/tsDMARD*

***Supplementary table 2:*** *Proportions of patients fulfilling the definitions A-E with the time restrictions, in the three nested populations (population (i)-(iii)), respectively.*

|  | Population (i) | Population (ii) | Population (iii) |
| --- | --- | --- | --- |
| Definition A1  Failure of at least two bDMARDs with different MoA. At least one TNFi and at least one non-TNFi. | 1.5 % (269) | 1.6 % (289) | 4.2 % (339) |
| Definition A2  Failure of at least two bDMARDs with different MoA. At least two non-TNFis. | 0.5 % (89) | 0.5 % (96) | 1.3 % (106) |
| Definition B1  Failure of at least three bDMARDs with different MOA. At least one TNFi AND two non-TNFis. | 0.36 % (64) | 0.37 % (66) | 1.0 % (81) |
| Definition B2  Failure of three non-TNFis. | 0.07 % (12) | 0.08 % (15) | 0.2 % (19) |
| Definition C  Failure of at least one TNFi and all available non-TNFis. | 0.07 % (12) | 0.08 % (14) | 0.2 % (19) |
| Definition D  Failure of at least one TNFi and all available non-TNFis PLUS at least one tsDMARD. | 0.03 % (6) | 0.04 % (7) | 0.09 % (7) |
| Definition E  Failure of ≥2 b/tsDMARDs with different MoA | 2.2 % (388) | 2.3 % (416) | 5.8 % (467) |

***Supplementary table 3:*** *Baseline^*^ characteristics of the definitions A-E in population (ii) and (iii).*

|  | Population (ii) | | | | | | | | | | | | Population (iii) | | | | | | | | | | | | | |
| --- | --- | --- | --- | --- | --- | --- | --- | --- | --- | --- | --- | --- | --- | --- | --- | --- | --- | --- | --- | --- | --- | --- | --- | --- | --- | --- |
| Definition | A1 | A2 | B1 | | B2 | | C | | D | | E | | A1 | | A2 | | B1 | | B2 | | C | | D | | E | |
| Age (years), mean (SD) | 53.0 (14.3) | 53.2 (13.8) | 51.6 (13.5) | | 49.5 (13.6) | | 48.5 (13.0) | | 45.4 (12.5) | | 53.2 (14.2) | | 53.0 (14.2) | | 53.1 (13.7) | | 51.7 (13.3) | | 49.6 (13.5) | | 48.7 (13.0) | | 46.4 (13.2) | | 53.1 (14.2) | |
| Male sex (%) | 22.7 | 22.8 | 23.1 | | 23.6 | | 24.0 | | 22.0 | | 22.8 | | 23.3 | | 22.7 | | 23.5 | | 19.3 | | 19.3 | | 20.3 | | 23.0 | |
| CRP, mg/L, median (IQR) | 10 (4–28) | 10 (4–26) | 10 (4–25) | | 13 (5–29) | | 13 (5–26) | | 10 (3–24) | | 10 (4-25) | | 8 (4–22) | | 9 (4–23) | | 9 (3–23) | | 10 (4–25) | | 10 (3–25) | | 9 (2–26) | | 8 (4-21) | |
| ESR, median (IQR) | 24 (12–41) | 23 (12–39) | 22 (12–38) | | 25 (14–42) | | 24 (14–39) | | 21 (12–36) | | 22 (12-40) | | 21 (11–36) | | 22 (11–37) | | 21 (11–36) | | 23 (12–39) | | 22 (12–36) | | 20 (10–36) | | 20 (10-36) | |
| PGA, VAS 0–100, mean (SD) | 57.9 (25.5) | 58.5 (25.3) | 58.9 (25.3) | | 58.4 (24.6) | | 58.4 (25.2) | | 62.0 (22.8) | | 57.1 (25.5) | | 57.9 (23.3) | | 60.0 (22.2) | | 60.2 (21.5) | | 62.2 (21.6) | | 62.0 (21.8) | | 63.5 (22.7) | | 57.4 (23.5) | |
| Pain, VAS 0–100, mean (SD) | 58.9 (25.6) | 58.9 (25.6) | 59.1 (25.5) | | 59.1 (25.1) | | 58.6 (25.6) | | 62.1 (23.7) | | 58.3 (25.7) | | 58.6 (24.0) | | 60.0 (23.5) | | 60.1 (22.9) | | 62.4 (22.7) | | 62.0 (22.0) | | 63.6 (22.7) | | 58.2 (24.1) | |
| Fatigue, VAS 0–100, mean (SD) | 59.8 (27.4) | 62.0 (27.2) | 62.2 (27.0) | | 59.1 (26.3) | | 59.1 (26.9) | | 64.4 (23.0) | | 58.9 (27.5) | | 59.3 (25.6) | | 62.3 (25.0) | | 62.3 (25.1) | | 64.7 (22.8) | | 65.2 (22.7) | | 66.9 (22.7) | | 58.5 (26.0) | |
| HAQ, mean (SD) | 1.13 (0.66) | 1.18 (0.65) | | 1.14 (0.63) | | 1.14 (0.63) | | 1.11 (0.57) | | 1.12 (0.56) | | 1.12 (0.66) | | 1.1 (0.6) | | 1.2 (0.6) | | 1.1 (0.6) | | 1.2 (0.6) | | 1.2 (0.6) | | 1.2 (0.5) | | 1.10 (0.63) |
| SJC, median (IQR) | 6 (3–10) | 5 (3–9) | | 5 (3–9) | | 5 (3–9) | | 5 (3–9) | | 4 (2–9) | | 6 (3-10) | | 5 (3–9) | | 6 (3–9) | | 6 (3–9) | | 6 (4–10) | | 6 (3–10) | | 6 (3–10) | | 5 (2-9) |
| TJC, median (IQR) | 7 (3–11) | 6 (3–11) | | 6 (3–11) | | 7 (4–11) | | 7 (4–11) | | 6 (4–11) | | 6 (3-11) | | 6 (3–11) | | 7 (4–11) | | 7 (4–11) | | 8 (4–13) | | 8 (4–14) | | 8 (4–13) | | 6 (3-10) |
| Concomitant csDMARD (%) | 85.2 | 82.5 | | 84.2 | | 82.7 | | 84.0 | | 89.8 | | 85.2 | | 55.5 | | 57.4 | | 57.8 | | 59.7 | | 62.4 | | 60.9 | | 72.3 |
| Concomitant glucocorticoids (%) | 55.9 | 57.7 | | 58.3 | | 60.0 | | 63.0 | | 64.4 | | 85.2 | | 55.5 | | 57.4 | | 57.8 | | 59.7 | | 62.4 | | 60.9 | | 72.3 |
| RF and/or ACPA pos (%) | 81.3 | 83.4 | | 83.3 | | 89.1 | | 89.0 | | 86.4 | | 79.1 | | 80.6 | | 82.4 | | 82.3 | | 89.1 | | 89.0 | | 84.4 | | 78.1 |
| DAS28-ESR, mean (SD) | 5.1 (1.4) | 5.0 (1.3) | | 5.0 (1.7) | | 5.2 (1.2) | | 5.1 (1.2) | | 5.1 (1.1) | | 5.0 (1.4) | | 4.9 (1.3) | | 5.1 (1.2) | | 5.1 (1.2) | | 5.2 (1.2) | | 5.2 (1.3) | | 5.2 (1.2) | | 4.9 (1.2) |
| *Comorbidities* |  | | | | | | | | | | | | | | | | | | | | | | | | | |
| Heart failure (%) | 0.7 | 0.6 | | 0.5 | | 0.0 | | 0.0 | | 0.0 | | 0.7 | | 1.1 | | 1.0 | | 0.8 | | 0.8 | | 0.9 | | 0.0 | | 1.1 |
| Ischemic heart disease (%) | 3.1 | 3.5 | | 3.5 | | 2.7 | | 3.0 | | 3.4 | | 2.7 | | 3.3 | | 4.5 | | 4.2 | | 2.5 | | 2.8 | | 3.1 | | 3.1 |
| Malignancy (%) | 1.8 | 2.3 | | 2.4 | | 1.8 | | 2.0 | | 1.7 | | 1.6 | | 2.0 | | 2.6 | | 2.3 | | 3.4 | | 3.7 | | 4.7 | | 2.0 |
| Renal failure (%) | 0.5 | 0.4 | | 0.5 | | 0.9 | | 1.0 | | 1.7 | | 0.4 | | 0.4 | | 0.3 | | 0.4 | | 0.8 | | 0.9 | | 1.6 | | 0.3 |
| COPD (%) | 1.8 | 2.1 | | 1.4 | | 0.9 | | 1.0 | | 0.0 | | 1.8 | | 1.5 | | 1.9 | | 1.0 | | 1.7 | | 1.8 | | 1.6 | | 1.7 |
| All respiratory (%) | 12.9 | 15.4 | | 11.8 | | 11.8 | | 10.0 | | 10.2 | | 14.2 | | 14.8 | | 18.2 | | 14.6 | | 19.3 | | 17.4 | | 18.8 | | 15.4 |
| Hospital infections (%) | 5.4 | 5.3 | | 4.5 | | 1.8 | | 1.0 | | 1.7 | | 5.4 | | 6.4 | | 7.3 | | 6.7 | | 3.4 | | 2.8 | | 3.1 | | 6.3 |
| Symptom duration before diagnosis, months, median (IQR) | 9 (4–42) | 11 (4–51) | | 10 (4–45) | | 8 (3–37) | | 8 (3–28) | | 6 (3–14) | | 9 (4-40) | | 11 (4–48) | | 12 (4–60) | | 12 (4–51) | | 9 (3–43) | | 9 (3–37) | | 6 (3–16) | | 11 (4-47) |
| Time from baseline until fulfilling definition, months, median (IQR) | 37 (18–62) | 26 (12–49) | | 52 (32–78) | | 60 (43–82) | | 61 (43–84) | | 61 (43–84) | | 53 (31-80) | | 37 (18–63) | | 24 (12–49) | | 52 (31–76) | | 60 (43–84) | | 60 (43–84) | | 80 (63–96) | | 53 (30-79) |
| Patient fulfilling definition within 5 years from baseline (%) | 6.0 | 2.4 | | 1.4 | | 0.3 | | 0.3 | | 0.2 | | 2.4 | | 14.6 | | 5.9 | | 3.4 | | 0.8 | | 0.7 | | 0.2 | | 5.8 |
| Patient fulfilling definition within 10 years from baseline (%) | 8.1 | 2.8 | | 2.3 | | 0.6 | | 0.5 | | 0.3 | | 4.0 | | 19.7 | | 7.1 | | 5.7 | | 1.5 | | 1.3 | | 0.8 | | 10.1 |

** (ii) start date first ever DMARD, (iii) start date first ever b/ts DMARD*

***Supplementary table 4:*** *Occurrence of patients fulfilling the different definitions of repeated discontinuation of b/tsDMARD with a restricted follow-up time of four years in each of the calendar time, in the three nested patient populations.*

| *Population (i)* | *Calendar time 2010-2014*  *% (N)* | *Calendar time 2015-2019*  *% (N)* |
| --- | --- | --- |
| *Definition A1* | 5.4 % (550) | 4.0 % (305) |
| *Definition A2* | 2.7 % (276) | 1.2 % (89) |
| *Definition B1* | 1.3 % (130) | 0.7 % (56) |
| *Definition B2* | 0.2 % (22) | 0.2 % (13) |
| *Definition C* | 0.2 % (21) | 0.2 % (11) |
| *Definition D* | 0.01 % (1) | 0.08 % (6) |
| *Definition E* | 1.8 % (178) | 1.8 % (137) |
| *Population (ii)* |  |  |
| *Definition A1* | 6.0 % (623) | 4.3 % (335) |
| *Definition A2* | 2.8 % (293) | 1.3 % (101) |
| *Definition B1* | 1.4 % (140) | 0.8 % (62) |
| *Definition B2* | 0.3 % (27) | 0.2 % (14) |
| *Definition C* | 0.2 % (23) | 0.2 % (12) |
| *Definition D* | 0.01 % (1) | 0.1 % (7) |
| *Definition E* | 1.7 % (192) | 2.1 % (160) |
|  |  |  |
| *Population (iii)* |  |  |
| *Definition A1* | 18.0 % (887) | 12.3 % (389) |
| *Definition A2* | 7.8 % (385) | 3.6 % (112) |
| *Definition B1* | 4.5 % (220) | 2.6 % (81) |
| *Definition B2* | 0.8 % (40) | 0.5 % (17) |
| *Definition C* | 0.7 % (35) | 1.4 % (44) |
| *Definition D* | 0.2 % (8) | 0.3 % (9) |
| *Definition E* | 6.7 % (331) | 6.0 % (188) |

***Supplementary table 5:*** Missing data in all three nested patient populations.

| Missing data | Population (i) | Population (ii) | Population (iii) |
| --- | --- | --- | --- |
| Age (yrs.), mean (SD) | 0 % | 0 % | 0 % |
| Sex (male), % | 0 % | 0 % | 0 % |
| CRP, mg/L, median (IQR) | 3.9 % | 3.7 % | 4.7 % |
| ESR, median (IQR) | 7.8 % | 7.4 % | 8.7 % |
| Pat global, VAS 0–100, mean (SD) | 12.3 % | 12.2 % | 9.6 % |
| Pat pain, VAS 0-100, mean (SD) | 13.8 % | 13.6 % | 10.4 % |
| Fatigue, VAS 0–100, mean (SD) | 49.6 % | 47.3 % | 24.2 % |
| HAQ, mean (SD) | 18.4 % | 18.3 % | 13.6 % |
| SJC, median (IQR) | 4.4 % | 3.8 % | 5.9 % |
| TJC, median (IQR) | 4.5 % | 3.9 % | 6.0 % |
| Concomitant csDMARD, % | 0 % | 0 % | 0 % |
| Concomitant GC, % | 0 % | 0 % | 0 % |
| RF and/or ACPA pos, % | 0 % | 0 % | 0 % |
| EGA, VAS 0-100, mean (SD) | 82.9 % | 81.2 % | 55.0 % |
| DAS28-ESR, mean (SD) | 19.9 % | 19.2 % | 17.4 % |
| *Comorbidities at baseline* |  |  |  |
| Heart failure, % | 0 % | 0 % | 0 % |
| Ischemic heart disease, % | 0 % | 0 % | 0 % |
| Malignancy, % | 0 % | 0 % | 0 % |
| Renal failure, % | 0 % | 0 % | 0 % |
| COPD, % | 0 % | 0 % | 0 % |
| All respiratory, % | 0 % | 0 % | 0 % |
| Hospital infections, % | 0 % | 0 % | 0 % |
| *Smoking status* | 67.8 % | 66.7 % | 39.3 % |
| Symptom duration before diagnosis, months, median (IQR) | 0.2 % | 0.2 % | 0 % |

***Supplementary table 6:*** *Information about comorbidity, up to 5 years before RA diagnosis until start of the observational period.*

| Heart failure | I50 | Patient Register, visits (main dx), hospitalization (main dx, but if main dx is M05* or M06* then bi diagnosis 1 is searched for the ICD 10 codes |
| --- | --- | --- |
| Ischemic heart disease | I20-I25 | Patient Register, visits (main dx), hospitalization (main dx, but if main dx is M05* or M06* then bi diagnosis 1 is searched for the ICD 10 codes |
| Malignancy excluding non-melanoma and basal cell invasive/non-invasive skin cancers | All non-benign tumors, except C44 and D04 (ICD7=191), and basal cell cancers | Cancer register |
| Renal failure | N18-19 | Patient Register, visits (main dx), hospitalization (main dx, but if main dx is M05* or M06* then bi diagnosis 1 is searched for the ICD 10 codes |
| COPD | J40-J44 |  |
| All respiratory tract diseases | J00-J99 |  |
| Hospitalized Infections | A00-B99, D73.3, E06.0, E32.1, G00-G02, G04.2, G05-G07, H00.0, H44.0, H60.0-H60.3, H66-H67, H70, I30.1, I40.0, J00-J22, J32, J34.0, J36, J38.3, J39.0-J39.1, J44.0, J85, J86, K04.4, K04.6, K04.7, K10.2, K11.3, K12.2, K14.0, K57.0, K57.2, K57.4, K57.8, K61, K63.0, K65.0, K65.1, K65.2, K65.9, L00-L08, L30.3, M00-M01, M46.2-M46.5, M60.0, M65.0, M71.0, M71.1, M72.6, M86, N10, N11, N12, N13.6, N15.1, N15.9, N30.0 N30.8, N34.0, N41.2, N43.1, N45.2, N45.3, N45.4, N48.2, N61, N70, N73, N75.1 | Main diagnosis in inpatient component of Patient Register. If main diagnosis was RA (ICD10 codes M05, M06.0, M06.2, M06.3, M06.8, M06.9, M12.3), contributory diagnoses of hospitalized infections were also allowed.  Note that infection data from non-primary (specialist) outpatient care was not included since it would be highly influenced by surveillance effects. In particular, patients seen for a chronic disease would in passing be more likely to also be recorded with these milder conditions. Regardless, most non-serious infections would not be treated in specialist care. |

***Supplementary table 7:*** *Proportions of patients per calendar period, fulfilling the definitions A-E, median time for fulfilment (months) and the cumulative incidence 5 years from respectively baseline.*

|  | Population (i) | | Population (ii) | | Population (iii) | |
| --- | --- | --- | --- | --- | --- | --- |
| Calendar time | 2010–2014 | 2015–2019 | 2010–2014 | 2015–2019 | 2010–2014 | 2015–2019 |
| Definition A1  Failure of at least two bDMARDs with different MoA. At least one TNFi and at least one non-TNFi. | 9.8 % | 4.7 % | 10.6 % | 5.0 % | 24.4 % | 13.1 % |
| Time fulfilling the definition, months, median (IQR) respectively cumulative incidence 5 years (%) | 43  (23–69),  6.5 % | 24  (13–39),  4.5 % | 43  (22–70),  4.0 % | 23  (13–39),  4.7 % | 43  (22–70),  1.9 % | 23  (13–37),  1.9 % |
| Definition A2  Failure of at least two bDMARDs with different MoA. At least two non-TNFis. | 3.8 % | 1.2 % | 3.9 % | 1.4 % | 9.3 % | 3.7 % |
| Time fulfilling the definition, months, median (IQR) respectively cumulative incidence 5 years (%) | 31  (13–53),  3.1 % | 16  (8–28),  1.2 % | 33  (13–55)  3.1 % | 16  (7–26),  1.4 % | 30  (13–54),  7.4 % | 16  (7–26),  3.6 % |
| Definition B1  Failure of at least three bDMARDs with different MoA. At least one TNFi AND two non-TNFis. | 3.1 % | 1.0 % | 3.3 % | 1.0 % | 7.9 % | 2.9 % |
| Time fulfilling the definition, months, median (IQR) respectively cumulative incidence 5 years (%) | 57  (37–80),  1.7 % | 35  (20–49),  0.9 % | 60  (36–84),  1.7 % | 32  (20–48),  0.9 % | 60  (35–84),  3.9 % | 41  (36–57),  1.7 % |
| Definition B2  Three non-TNFis | 0.8 % | 0.2 % | 1.1 % | 0.2 % | 2.0 % | 0.7 % |
| Time fulfilling the definition, months, median (IQR) respectively cumulative incidence 5 years (%) | 67  (48–89),  0.3 % | 41  (29–57),  0.2 % | 61  (43–84),  0.5 % | 41  (29–50),  0.2 % | 65  (46–88),  0.8 % | 41  (33–57),  0.6 % |
| Definition C  Failure of at least one TNFi and all available non-TNFis | 0.7 % | 0.2 % | 1.0 % | 0.2 % | 1.8 % | 0.7 % |
| Time fulfilling the definition, months, median (IQR) respectively cumulative incidence 5 years (%) | 67  (47–88),  0.3 % | 43  (35–58),  0.2 % | 61  (43–84),  0.5 % | 43  (35–55),  0.2 % | 66  (46–88),  0.8 % | 41  (36–57),  0.6 % |
| Definition D  Failure of at least one TNFi and all available non-TNFis PLUS at least one tsDMARD | 0.4 % | 0.1 % | 0.6 % | 0.1 % | 1.1 % | 0.4 % |
| Time fulfilling the definition, months, median (IQR) respectively cumulative incidence 5 years (%) | 85  (74–99),  0.3 % | 40  (33–59),  0.1 % | 79  (63–90),  0.1 % | 42  (34–58),  0.1 % | 84  (73–100),  0.06 % | 38  (34–55),  0.3 % |
| Definition E  Failure of ≥2 b/ts DMARDs with different MoA | 12.1 % | 6.8 % | 12.8 % | 7.4 % | 29.3 % | 19.1 % |
| Time fulfilling the definition, months, median (IQR) respectively cumulative incidence 5 years (%) | 63  (39–89),  0.03 % | 32  (20–48),  2.1 % | 66  (40–90),  1.3 % | 31  (19–46),  1.0 % | 65  (38–90),  5.6 % | 31  (18–47),  6.0 % |
